# Supplementary material for: Identification of Antioxidant Peptides Derived from Tilapia (Oreochromis niloticus) Skin and Their Mechanism of Action by Molecular Docking
Source: Foods. 2022 Aug 25;11(17):2576. doi: 10.3390/foods11172576 (PMC9455858; doi:10.3390/foods11172576)
Supplement: Supplementary file 1 [file foods-11-02576-s001.zip › foods-1853124-supplementary.pdf]

Supporting Information

Identification of Antioxidant Peptides Derived from Tilapia (*Oreochromis niloticus*) Skin and Their Mechanism of Action by Molecular Docking

Yueyun Ma [1], Dandan Zhang [1], Mengqi Liu [1], Yingrou Li [1], Rui Lv [1], Xiang Li [1,2,3,4], Qiukuan Wang [1,2,3,4], Dandan Ren [1,2,3,4], Long Wu [1,2,3,4], and Hui Zhou [1,2,3,4,*]

[1] College of Food Science and Engineering, Dalian Ocean University, Dalian 116023, China
[2] National R&D Branch Center for Seaweed Processing, Dalian 116023, China
[3] Key Laboratory of Aquatic Product Processing and Utilization of Liaoning Province, Dalian 116023, China
[4] Collaborative Innovation Center Jointly Established by Ministries and Ministries for Key Technologies of Deep Processing of Marine Food, Dalian Polytechnic University, Dalian 116034, China
* Correspondence: zhouhui@dlou.edu.cn

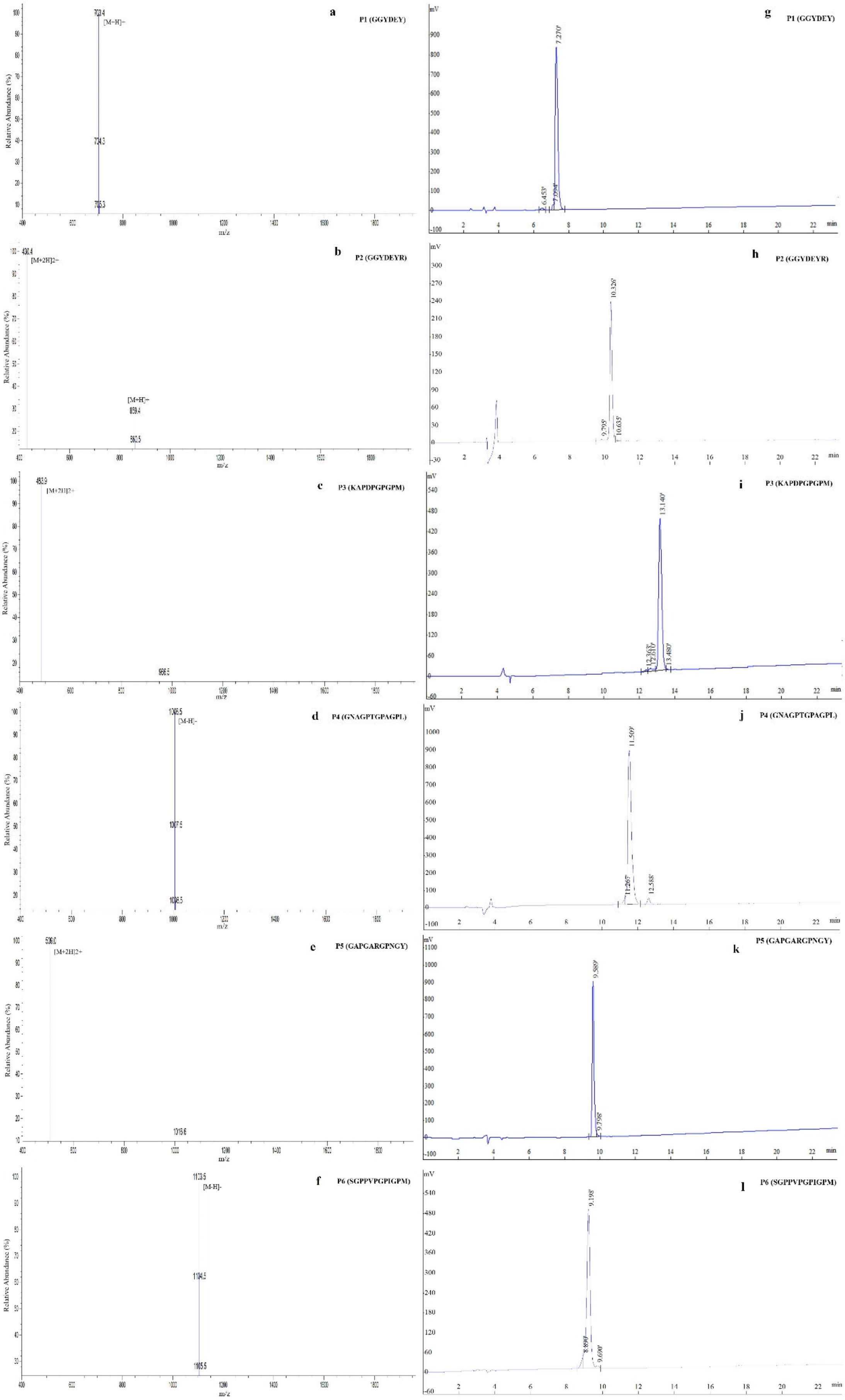

a
P1 (GGYDEY)
[M-H]−
b
P2 (GGYDEYR)
[M+2H]2−
[M+H]+
c
P3 (KAPDPGPGPM)
[M+2H]2+
d
P4 (GNAGPTGPAGPL)
[M-H]-
e
P5 (GAPGARGPNGY)
[M+2H]2+
f
P6 (SGPPVPGPIGPM)
[M-H]-
Relative Abundance (%)
m/z
g
P1 (GGYDEY)
7.270
h
P2 (GGYDEYR)
10.326
i
P3 (KAPDPGPGPM)
13.140
13.480
j
P4 (GNAGPTGPAGPL)
11.509
11.267
12.588
k
P5 (GAPGARGPNGY)
9.585
9.798
l
P6 (SGPPVPGPIGPM)
9.198
mV
min

Figure S1. MS and HPLC characterization spectra of synthetic antioxidant peptides. (**a-f**) MS spectra of P1-P6; (**g-l**) HPLC spectra of P1-P6. MS test conditions: Instrument: Agilent-6125B; Probe: ESI; Probe Bias: ＋4.5kv; Nebulizer Gas Flow: 1.5L/min; Detector: 1.5kv; CDL: -20.0v; T. Flow: 0.2ml/min; CDL Temp.:250℃; A. Conc.: 100%H2O; B. Conc.: 50%H2O/50%ACN; Block Temp.: 200℃. HPLC test conditions: Column: Gemini-NX 5μ C18 (110A, 4.6*250mm); Solvent A :0.1% TFA in 100% CAN; Solvent B : 0.1% TFA in 100% Water; Elution gradient: 0–25 min, 85–60% eluent B; 25-25.01 min, 60–0% eluent B; Flow rate:1.0ml/min; Wavelength:220nm; Volume:20ul .
